# Influence of activating Fcgamma receptors on osteoclast differentiation and bone metabolism

**DOI:** 10.1186/1479-5876-10-S3-P24

**Published:** 2012-11-28

**Authors:** Michaela Seeling, Jan Peter Tuckermann, Jean Pierre David, Georg Schett, Falk Nimmerjahn

**Affiliations:** 1 Dept. of Biology, University Erlangen-Nuremberg, Erlangen, Germany; 2 Institute of General Zoology and Endocrinology, University of Ulm, Ulm, Germany; 3 Dept. of Osteology and Biomechanics, University Medical Center Hamburg-Eppendorf, Hamburg, Germany; 4 Dept. of Internal Medicine 3, University of Erlangen-Nuremberg, Erlangen, Germany

## Background

Patients with Rheumatoid Arthritis suffering from chronic inflammation of the joints have a higher risk of developing osteoporosis. One hallmark of this inflammatory process is an increased production of autoantibodies. It is unclear, whether the autoantibodies can act directly on cells of the bone metabolism via Fcgamma receptors (FcγRs). Thus, the aim of this project is to analyze the influence of the activating FcγR expression on osteoclasts, the bone-resorbing cells, and the bone metabolism *in vitro* and *in vivo* by comparison of C57Bl/6 and several FcγR knockout mice.

## Materials and methods

FACS analysis of *in vitro* cultivated osteoclasts was performed to confirm the expression of FcγRs. Their influence on the differentiation of bone marrow cells to mature osteoclasts was analyzed by crosslinking the receptors during the cultivation. To simulate a rheumatoid arthritis *in vivo* the KRN-serum transfer model was used. Assessment of the inflammatory effects was performed by serological, structural and histomorphomic analysis.

## Results

FACS analysis showed that i*n vitro* cultivated osteoclasts upregulated the activating FcγRs during differentiation. The maturation process of osteoclasts was positively influenced upon crosslinking of the FcγRI and IV *in vitro*. In healthy mice and in mice suffering from rheumatoid arthritis different effects of the activating FcγRs was observed. Also, osteoclasts in healthy FcγRIV knockout mice tended to be smaller. During a rheumatoid arthritis the loss of the FcγRI and IV protected from inflammatory bone loss, although apparently by different mechanisms.

## Conclusions

Our findings show that autoantibodies, which occur in increased amounts during the chronical inflammation of a rheumatoid arthritis, can have a direct effect on the bone-resorbing osteoclasts via binding on FcγRs expressed on their surface.

